# Cell-in-cell structures are more potent predictors of outcome than senescence or apoptosis in head and neck squamous cell carcinomas

**DOI:** 10.1186/s13014-016-0746-z

**Published:** 2017-01-18

**Authors:** Hannah Schenker, Maike Büttner-Herold, Rainer Fietkau, Luitpold V. Distel

**Affiliations:** 1 Department of Radiation Oncology, Universitätsklinikum Erlangen, Friedrich-Alexander-Universität Erlangen-Nürnberg (FAU), Universitätsstraße 27, 91054 Erlangen, Germany; 2 Department of Nephropathology, Institute of Pathology, Universitätsklinikum Erlangen, Friedrich-Alexander-Universität Erlangen-Nürnberg (FAU), Krankenhausstr. 12, 91054 Erlangen, Germany

**Keywords:** HNSCC, Cell death, Cell-in-cell, Senescence, Apoptosis, Proliferation

## Abstract

**Background:**

This study sheds light on cell inactivating processes with focus on the phenomenon of cell-in-cell (CIC). Cell-in-cell describes a cell process where one cell is being engulfed by another non-professional phagocyte. We determined frequency and prognostic impact of CIC structures (CICs) as well as of senescent and apoptotic cells in head and neck squamous cell carcinomas (HNSCC).

**Methods:**

These different forms of cell inactivation as well as the proportion of proliferating and tumor cells were assessed in 169 pre-radiochemotherapy biopsies and 32 post-therapy tumor resections by immunohistochemistry of tissue microarrays. Four consecutive cancer sections were stained with antibodies specific for E-cadherin for CIC detection, cleaved caspase-3 for apoptosis, H3K9Me for senescence and Ki67 as a proliferation marker. Positive events were quantified in corresponding tumor areas.

**Results:**

CICs were found in 55.5%, senescent cells in 67.1% and apoptotic cells in 93.3% of samples. While no prognostic impact of apoptotic and senescent cells was observed, CICs turned out to significantly influence overall-survival (*p* = 0.016) with a lack of CICs being prognostically beneficial. There was no correlation between CICs and apoptosis and 98.9% of CICs were negative for cleaved caspase-3.

**Conclusion:**

CIC formation is a frequent event in HNSCC and a superior predictive marker compared to senescence and apoptosis. Independence of CIC and apoptosis and the adverse prognosis associated with numerous CICs lead to the assumption that CICs might take up necrotic rather than apoptotic cells preventing an adequate antitumoral immune response that would otherwise be initiated by necrotic cells through damage-associated molecular pattern molecules.

**Electronic supplementary material:**

The online version of this article (doi:10.1186/s13014-016-0746-z) contains supplementary material, which is available to authorized users.

## Background

In multicellular organisms, precise control and coordination of cell proliferation, cell inactivation and cell death are vital to maintain homeostasis of cells in tissues and organs as slightest imbalances can lead to pathologies like tumors and autoimmune diseases. For deeper understanding of these processes and their meaning we investigated the cell processes of cell-in-cell, senescence and apoptosis. Much attention has been focused on the cell death mechanisms of apoptosis and necrosis and their significance in tumors as well as healthy tissue. However, the far less appreciated cell inactivating processes of CIC and senescence may be equally essential. To assess their role with regard to frequencies in tumor specimens and clinical outcome we investigated a cohort of HNSCC.

To best look into the subject of cell death stringent definitions are vital. Kroemer et al. suggest that cell death can be classified according to morphology, enzymological criteria, functional aspects or immunological characteristics [1]. The Nomenclature Committee on Cell Death (NCCD) previously proposed three criteria for the identification of a dead cell: (1) permanent loss of the barrier function of the plasma membrane; (2) breakdown of cells into discrete fragments; or (3) engulfment of cells by professional phagocytes or other cells endowed with phagocytic activity [2].

According to the NCCD the phenomena of apoptosis and necrosis can be further defined as follows: apoptosis is characterized by cytoplasmic shrinkage, chromatin condensation (marginalization), nuclear fragmentation (karyorrhexis), so called blebbing and apoptotic bodies and is considered a “regulated cell death”, generally referred to as “programmed cell death”. Necrosis presents generalized swelling of the cytoplasm and organelles (oncosis), alteration of chromatin (condensation) and the nuclear membrane (dilatation) and is regarded as “accidental cell death” [1]. Apart from these morphological features there was no reliable marker for detection of necrosis available.

Senescence defines the process of a cell going into an irreversible cell-cycle arrest that is unresponsive to mitogenic or oncogenic stimulation. However, senescent cells are still viable and metabolically active without displaying the specific functions of their lineage [3]. The CIC phenomenon describes a cell process where one cell is being phagocytized completely by another non-professional phagocytizing cell which has been observed in a variety of malignancies. “Cell-in-cell” is an umbrella term without further specification. Similar, yet different, processes giving rise to cell-in-cell structures have been introduced in literature: entosis, emperipolesis, cannibalism and phagocytosis [4]. Entosis is the active invasion of a living cell into another cell’s cytoplasm [5]. Emperipolesis defines the interaction of lymphocytes with other cells and has been observed in physiological and pathophysiological settings [6]. Cannibalistic tumor cells are able to engulf other cells, including lymphocytes and erythrocytes, either dead or alive, with the main purpose to feed on them [7]. These mechanisms all have in common that a living cell is taken up by another non-professional phagocytizing cell and can be broadly characterized as heterotypic or homotypic [8]. Although it is shown that an engulfed cell is able to remain in the host cell, evade the host cell or undertake cell division, phagocytosis most likely leads to cell death of the incorporated cell and a subsequent decomposition [9, 10]. Although the CIC phenomenon has recently gained more and more attention, so far exact details of its characteristics and mechanisms remain unclear. Preliminary findings originate several questions: Which are role and condition of engulfing and inner cell? Is the inner cell viable or dead? Is cell-in-cell a kind of cell death or rather a cell interaction process?

Our main focus in this study was to examine the formation of CIC as a specific form of non-professional phagocytosis. We assessed CIC’s prognostic meaning to further examine its association with clinical pathogenesis as well as its relation to apoptosis and senescence to gain further knowledge about the identity of the interacting cells. We quantified the frequencies of each of these events in a HNSCC cohort pre- and post-radiochemotherapy (RCT) and investigated their impact on local failure-free-, metastasis-free- and overall-survival.

## Materials and methods

### Human specimens

**Fig. 1:**
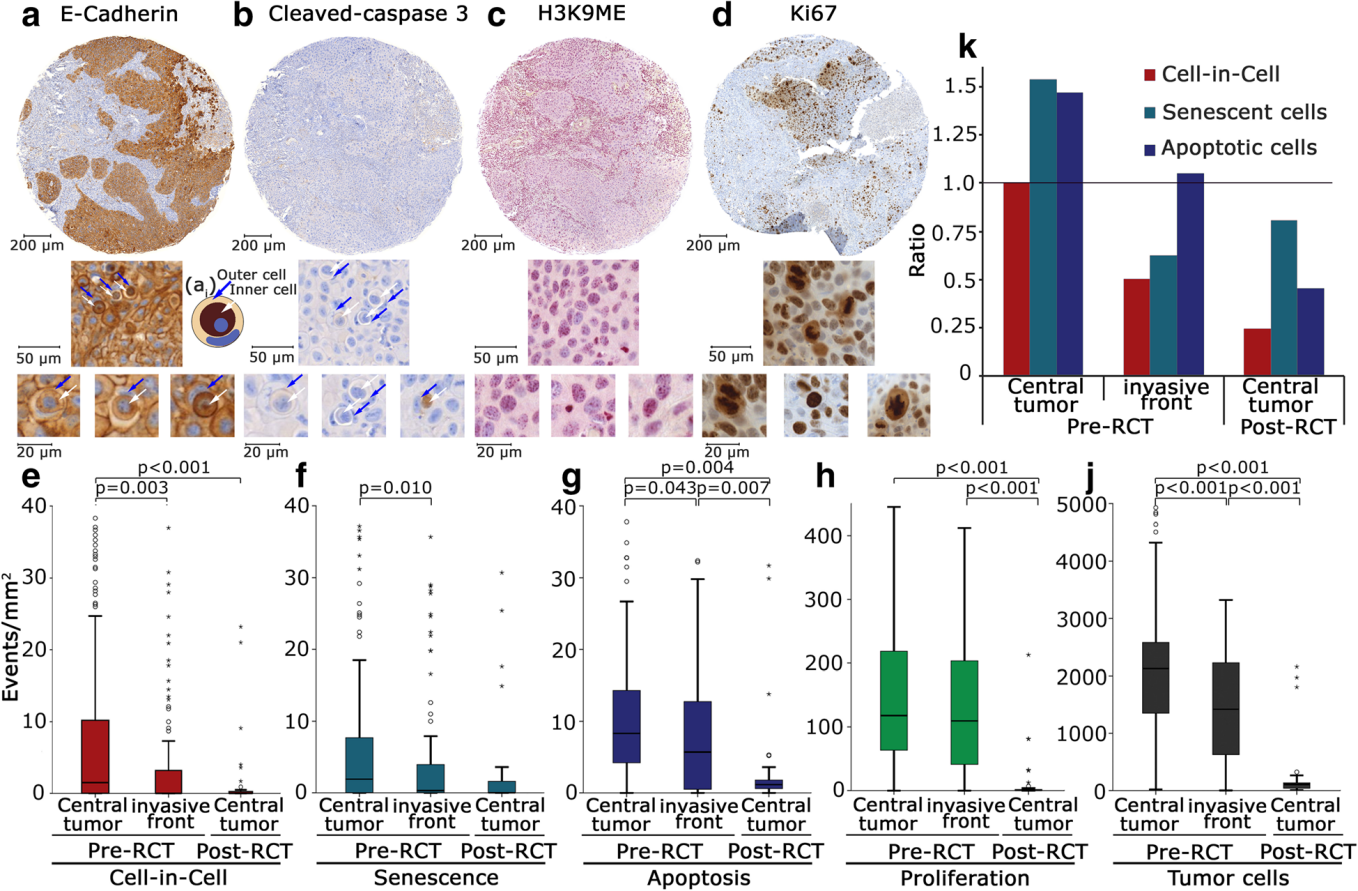
Frequency of CIC structures, senescent and apoptotic cells assessed by immunohistochemistry. **a** E-Cadherin staining labelling numerous CIC structures in HNSCC and magnifications of indicated region. (a_i_) Schematic drawing of a CIC structure illustrating defining criteria: complete encirclement of the inner cell by the host cell membrane, circular shape of the inner cell and semilunar host cell nucleus. **b** Cleaved caspase-3 labeled HNSCC and magnifications of indicated region showing CIC structures negative for cleaved caspase-3. **c** H3K9ME labeled HNSCC showing senescent cells and magnifications of indicated region. **d** Ki67 labeled HNSCC showing proliferating cells and magnifications of indicated region. Comparative analysis of (**e**) CIC structures, (**f**) senescent cells, (**g**) apoptotic cells, (**h**) proliferating cells and (**j**) tumor cells in the center of the tumor (pre- and post-RCT) and invasive front (pre-RCT). **k** Comparison of ratio of CIC structures, senescent cells and apoptotic cells in the center of the tumor and invasive front compared to frequency of CIC structures in the center of the tumor pre-RCT as a reference

Cancer tissue samples of 169 patients with HNSCC were evaluated including 32 post-therapeutic biopsies (Table 1). The post-therapy specimens were collected after a minimum of 12 days, a maximum of 379 days and an average of 46 days. The cohort consists of 29 female and 140 male patients with a median age of 56.4 years. All TNM stages are represented and tumor localizations include oropharynx, floor of the mouth and alveolar ridge. Patients were treated by definite, adjuvant or neoadjuvant radiotherapy or radiochemotherapy. Additional patients’ characteristics are shown in Table 1 as well as characteristics for patients with post-therapeutic samples. All samples were processed into tissue microarrays (TMAs) with cores of 1.6–2 mm diameter (Fig. 1a-d). There were 2–4 available TMA cores per patient. Appendant clinical data were extracted from the Erlangen Tumor Center Database and from the patients’ records. Pretherapeutic biopsies from the center of the tumor and the invasive front as well as post-therapy tumor resections were separately evaluated and compared. All patients signed a “front door” informed consent allowing collection of their tissue and clinical data. The study was approved by the Ethics Review Committee of the University Hospital Erlangen-Nürnberg, Erlangen, Germany.

**Table 1:** Clinical characteristics of the head and neck squamous cell carcinoma cohort

169 patients with available pre-RCT biopsies	
Gender	
Male	140 (82.8%)
Female	29 (17.2%)
Age	<56.4: 85 (50.3%) >56.4: 84 (49.7%)
T-Stage	
T1	38 (24.2%)
T2	58 (36.9%)
T3	29 (18.5%)
T4	32 (20.4%)
N-Stage	
N0	33 (21%)
N1	48 (30.6%)
N2	69 (43.9%)
N3	7 (4.5%)
M-Stage	
M0	132 (98.5%)
M1	2 (1.5%)
Grading	
G1	9 (5.7%)
G2	90 (56.6%)
G3	60 (37.7%)
Localization of tumor	
Oropharynx	83 (49.1%)
Base of the mouth	79 (46.8%)
Alveolar ridge	7 (4.1%)
Radiotherapy	
Neoadjuvant	32 (18.9%)
Adjuvant	137 (81.1%)
Mean total dose	59.2 Grey
Mean dose per fraction	2.0 Grey
Chemotherapy	
None	49 (29%)
5-FU + Cisplatin	93 (55%)
5-FU + Carboplatin	9 (5.3%)
Others	18 (10.7%)
32 patients with available post-RCT biopsies	
Gender	
Male	28 (87.5%)
Female	4 (12.5%)
Age	<51.4: 71 (46.4%) >51.4: 82 (53.6%)
T-stage	
T1	2 (6.3%)
T2	10 (31.2%)
T3	8 (25%)
T4	12 (37.5%)
N-Stage	
N0	11 (34.4%)
N1	21 (65.6%)
M-Stage	
M0	32 (100%)
Grading	
G1	1 (3.7%)
G2	21 (77.8%)
G3	5 (18.5%)
Localization of tumor	
Oropharynx	8 (25.0%)
Base of the mouth	23 (71.9%)
Alveolar ridge	1 (3.1%)
Radiotherapy	
Neoadjuvant	32 (100%)
Mean total dose	50.1 Grey
Mean dose per fraction	1.8 Grey
Chemotherapy	
None	1 (3.1%)
5-FU + Cisplatin	29 (90.6%)
5-FU + Carboplatin	2 (6.3%)

### Antibodies and immunohistochemistry

The following antibodies were used: anti-E-cadherin (1:2000; clone 36/E-Cadherin; BD, Heidelberg, Germany) to visualize membranes of cells to detect CIC structures (Fig. 1a), anti-cleaved caspase-3 (1:200, polyclonal; Cell Signaling, Danvers, MA, USA) to display apoptotic cells (Fig. 1b), anti-H3K9Me (1:200; [BM1] Millipore, Darmstadt, Germany) to detect senescent cells by chromatin condensation (Fig. 1c) and anti-Ki67 (1:100, clone MIB1; Dako Cytomation, Hamburg, Germany) to highlight proliferating cells (Fig. 1d). Hematoxylin-eosin staining was used for nuclear counter-staining. H3K9Me was stained manually using a steam cooker and a commercially available target retrieval solution pH 6 (TRS6, Dako Cytomation, Hamburg, Germany) for antigen retrieval and an alkaline phosphatase-labeled polymer kit (Zytochem-Plus AP-PolymerKit, Zytomed Systems, Berlin, Germany) and Fast Red (Sigma-Aldrich, Deisenhofen, Germany) for detection. The other stainings were performed on a Ventana BenchMark ULTRA stainer (Roche) using an UltraView DAB IHC Detection Kit (Roche) for detection.

### Imaging and image analysis

Stained TMAs were scanned with a high throughput scanner (Mirax Scan, Zeiss, Göttingen, Germany). The software Pannoramic Viewer (3D Histech, Budapest, Hungary) was used for further processing of the scans. Each TMA image was cut, exported and converted into TIF files to be saved separately. Cell events were counted in the same tumor area by precise alignment of each stained TMA using the image processing software Biomas (Erlangen, Germany). Each region of interest was defined in the E-cadherin slide and transferred to corresponding TMAs. Subsequently CICs, senescent cells, apoptotic cells, proliferating cells as well as tumor cells were counted partly manually partly half-automatically in each TMA by the use of the above-mentioned image processing software.

### Evaluation criteria

The following three criteria were defined for a coherent evaluation of CICs: complete encirclement of the inner cell by the host cell membrane, a round shape of the inner cell and a semilunar host cell nucleus displaced to the margin of the host cell (Fig. 1a_i_).

### Statistical analysis

IBM SPSS Statistics version 19 (IBM Corp, Armonk, NY, USA) was used for statistical analysis. Local failure-free survival, metastasis-free-survival and overall survival were determined according to Kaplan Meier. The log-rank test was used to compare survival curves between subgroups of patients. *P*-values below 0.05 were considered significant.

## Results

### Frequency of cell events

Clinical characteristics of the study group are given in Table 1. The HNSCC cohort consisted of 169 patients with pre-RCT biopsies from all 169 patients and post-RCT tissue samples from 32 patients. The median follow-up was 4.5 years. Four consecutive cancer sections were stained with E-cadherin for CIC detection (Fig. 1a), anti-cleaved caspase-3 for detection of apoptosis (Fig. 1b), anti-H3K9Me for detection of senescence (Fig. 1c) and anti-Ki67 for identification of proliferating cells (Fig. 1d). Apoptosis, senescence and proliferation were assessed in positive cells morphologically consistent with carcinoma cells, whereas other cells like stromal or inflammatory cells were not included in the analysis.

We compared the frequency of CICs to senescent, apoptotic, proliferating and tumor cells (Fig. 1e-j). Center of the tumor pre-RCT (*n* = 169), invasive front pre-RCT (*n* = 103) and center of the tumor post-RCT (*n* = 32) derived 6 weeks after the end of RCT were separately evaluated and compared with each other. Significantly more CICs, senescent cells and apoptotic cells were identified in samples from tumor centers compared to invasive front samples (*p* = 0.003, *p* = 0.010, *p* = 0.043) (Fig. 1e-g). Average numbers of all positive events as well as the proportion of samples showing any positive event in all respective markers decreased distinctly post-RCT. The highest number of proliferating cells was found in the center of the tumor with distinct decrease post-RCT (Fig. 1h). Additionally, tumor cells in each TMA were counted (Fig. 1j).

### Distribution and ratio of cell events

**Fig. 2:**
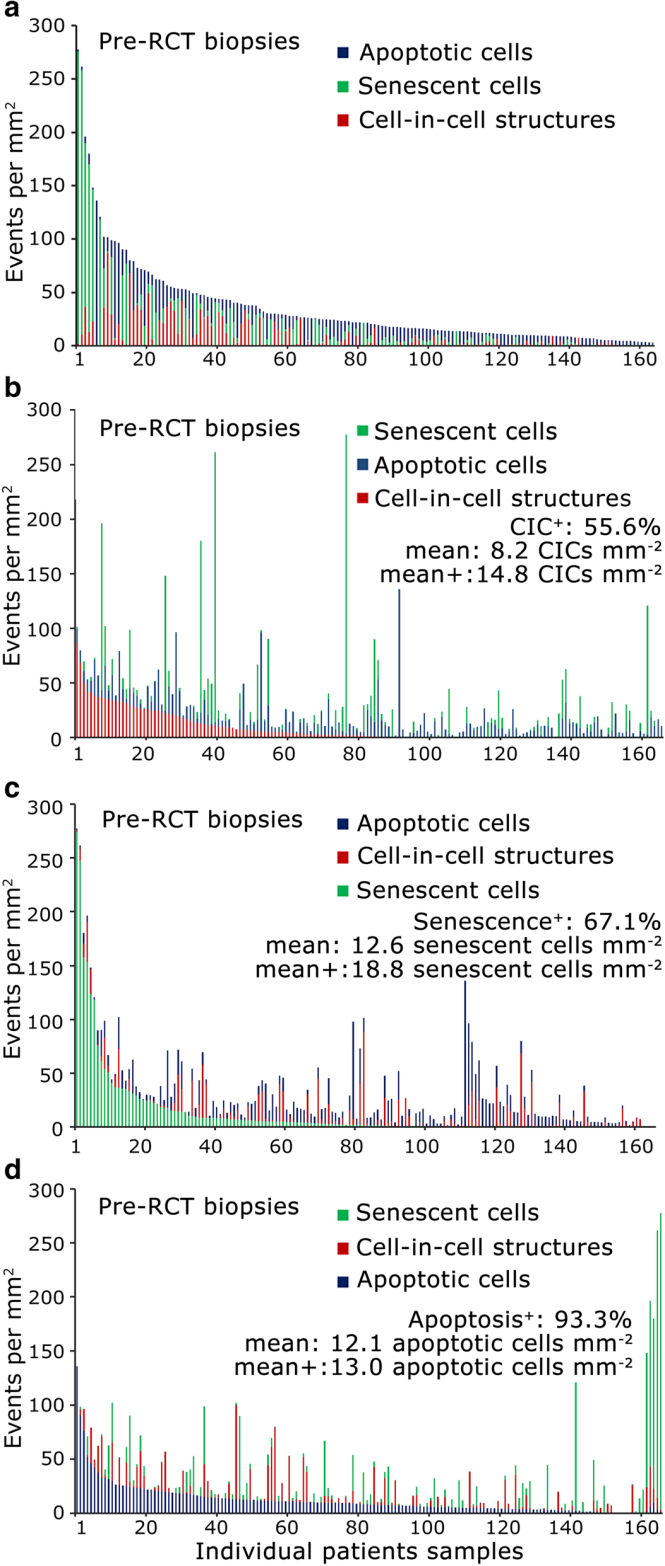
Frequency and distribution of CIC structures, senescent and apoptotic cells. **a** Overall frequency of CIC, senescent cells and apoptotic cells in pre-therapeutic biopsies. Distribution of cell events arranged in order of frequency of (**b**) CIC, (**c**) senescence and (**d**) apoptosis. The percentage of tissue samples having positive events, the overall mean (mean) and mean of tumor tissue with positive events only (mean+) is indicated

Subsequently the ratio of senescence, apoptosis and CIC was determined in relation to CICs in the center of the tumor as a reference (Fig. 1k). In the center of the tumor of pre-RCT biopsies senescent cells constitute the major fraction, whereas apoptotic events predominated in the invasive front. Senescent cells were the most common events post-RCT while apoptotic cells decrease. Figure 2a depicts the overall distribution of each event. CICs were found in 55.6% of the investigated cases (Fig. 2b), senescent cells were detected in 67.1% of tissue samples (Fig. 2c) and apoptotic cells in 93.3% (Fig. 2d). Numbers of CIC structures varied from 0 to 86.7 cells mm^−2^, senescent cells from 0 to 273.8 mm^−2^ and apoptotic cells from 0 to 136 cells mm^−2^.

Taking all tissue samples into consideration, nearly every biopsy showed apoptotic events while only about half display CICs and senescent cells each. However 84.6% of tissues were positive for either CICs or senescent cells. When looking at cases with events, absolute numbers of cases with CICs and senescent cells were comparably frequent as cases with apoptotic cells. The overall mean of CICs was 8.2 CICs mm^−2^ and rising to 14.8 CICs mm^−2^ when considering only CIC-positive samples (Fig. 2b). Accordingly, overall mean of senescent cells was 12.6 mm^−2^ and increased to 18.8 mm^−2^ after exclusion of negative cases (Fig. 2c). The mean value of apoptotic cells was only slightly changed when considering positive cases only as apoptotic events were present in the great majority of cases from the beginning (Fig. 2d).

### Correlation of cell events

**Fig. 3:**
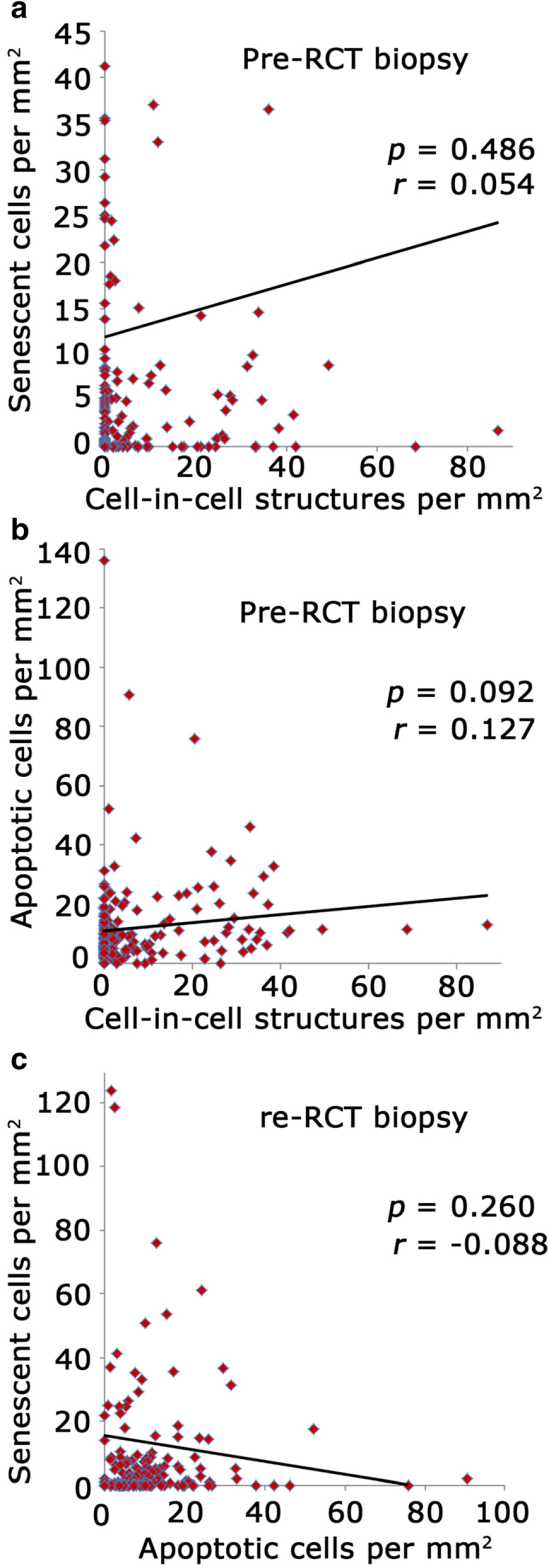
Correlation analysis. Correlation between (**a**) CIC and senescence, (**b**) CIC and apoptosis and (**c**) apoptosis and senescence

To assess whether there is a relation between the frequencies of CIC structures, senescence and apoptosis, we performed correlation analysis of all three events. The correlation between the three events was only weak and did not reach statistical significance (Fig. 3a-c). We further considered whether the internalized cells of CICs are apoptotic cells. To answer this question we investigated tumor areas in which CICs were found in corresponding regions in the cleaved caspase-3 staining. In only 16 out of 1466 CIC structures apoptotic cells were detected, indicating that 98.9% of CICs were negative for cleaved caspase-3 and therefore apparently not involving phagocytosis of apoptotic cells. Figure 1b illustrates an example of a clearly detectable CIC structure negative for cleaved caspase-3. We thereby presume that CIC formation is a phenomenon of non-apoptotic phagocytosis.

### Prognostic relevance of CIC structures, senescence and apoptosis

**Fig. 4:**
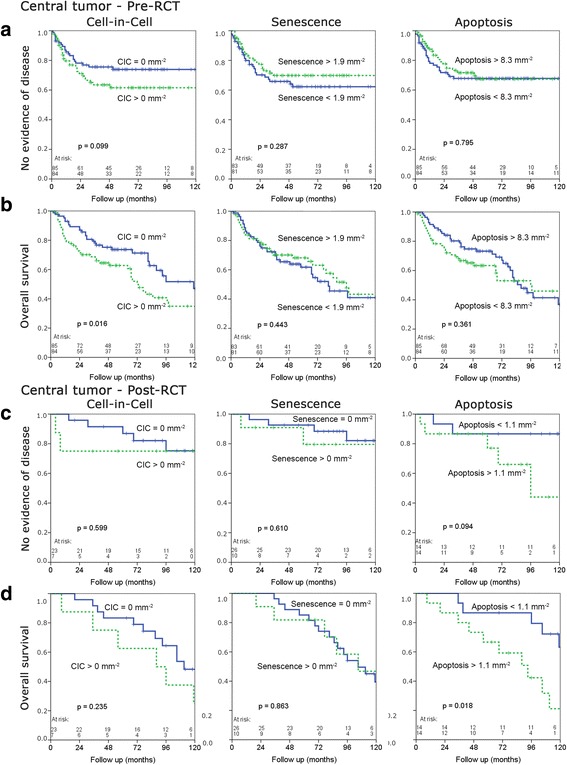
Kaplan-Meier analyses. Influence of CIC structures per mm^2^, senescent cells per mm^2^ and apoptotic cells per mm^2^ in the center of the tumor of pre-therapeutic biopsies on NED (**a**) and overall survival (**b**). Influence of CIC structures per mm^2^, senescent cells per mm^2^ and apoptotic cells per mm^2^ in the center of the tumor of post-therapeutic biopsies on NED (**c**) and overall survival (**d**). The median served as a cut off to separate group in all analyses. Blue solid lines indicate cases below the median and green spotted lines with counts over the median

Survival rates were calculated according to Kaplan Meier (Fig. 4a-d). CIC formation turned out to be a highly significant predictor of overall-survival in pre-therapeutic biopsies (*p* = 0.016), whereby a lack of CICs was beneficial for the patients (Fig. 4b). CICs also have the highest impact on failure-free survival (Additional file 1: Figure S1a) and metastasis-free survival (Additional file 1: Figure S1b) in pre-therapeutic biopsies of the central tumor. Occurrence of senescent cells was associated with a trend towards a prognostic advantage in pre-therapeutic biopsies (Fig. 4b). The presence or absence of apoptotic cells did not influence outcome pre-RCT (Fig. 4a, b). In post-RCT biopsies, the prognostic significance of CICs was lost compared to pre-RCT biopsies (Fig. 4c, d). However, apoptotic cells had a distinct impact on overall survival (*p* = 0.018) in post-RCT tissues (Fig. 4d) with no or little apoptotic events being prognostically favorable. Apoptotic cells also show prognostic significance regarding local failure-free survival (*p* < 0.001) and no evidence of disease (NED) (*p* < 0.001) post-RCT (Additional file 1: Figure S1c). Kaplan Meier analysis of proliferating cells and number of tumor cells are not depicted as they showed no prognostic value.

## Discussion

Our observations on frequency and prognostic impact of CICs, senescence and apoptosis evoke pivotal questions in view of previous findings and require further reflection. A remarking result is the fact that CICs and senescence occur in a comparable frequency range as apoptosis while not receiving equal consideration in literature. It is particularly noteworthy that CICs compared to apoptotic cell death, inactivation by senescence and proliferation is the only event with a prognostic relevance pre-RCT. Necrotic death could not be evaluated in this study due to the lack of a suitable immunohistochemical necrosis marker.

Another interesting point is that the prognostic value of CICs post-RCT was weaker than pre-RCT. Similarly, apoptosis showed no prognostic value pre-RCT whereas low apoptotic rates were associated with a favorable prognosis post-RCT. RCT-driven selection processes may lead to this change. The fraction of CIC-positive tissues post-RCT consisted of only 23%, while 50% of tissues were CIC-positive prior to RCT. Median apoptotic rates changed from 8.3 apoptotic cells per mm^2^ prior to RCT to 1.1 apoptotic cells per mm^2^ post-RCT.

As only very few of the cells engulfed in CICs were apoptotic and there was no significant correlation between CICs, apoptotic and senescent cells, the question rises whether CICs are formed by phagocytized necrotic cells. In our previous study 99.5% of CICs were negative for cleaved caspase-3 going in line with the present finding that apoptotic cells are very infrequently involved in the formation of CICs. There was also no correlation between these events, indicating that high numbers of apoptotic cells do not promote CIC formation [10]. Additionally we demonstrated in our previous studies that phagocytosis by nonprofessional phagocytes including primary fibroblasts, pancreatic adenocarcinoma, glioblastoma and head and neck cancer cells serves as a mechanism to remove primary necrotic cells [11]. While our data are consistent with a non-apoptotic cell-in-cell process they do not rule out other scenarios previously described in literature. For instance, Huang et al. identified four subtypes of CICs which could be formed homotypically among tumor cells or heterotypically between tumor cells and leukocytes. They also stated that macrophages could target live tumor cells to generate CICs [12]. As mentioned above, entosis is described a nonapoptotic cell death program in matrix-detached cells initiated by the invasion of one living cell into another [5]. Wang et al. report “emperitosis”, an apoptotic cell-in-cell death process that occurs in heterotypic immune killer cells expressing granzyme B inside tumor cells which may serve as an in-cell danger sensation model to prevent the killing of target cells from inside, implying a unique mechanism for tumor cells to escape from immune surveillance [13].

In our study, CICs turned out highly significant for overall-survival whereby high CIC occurrences were associated with poor survival rates. Going in line with other published data, this may support the possibility of CIC formation stimulating tumor survival and progression by inducing aneuploidy and promoting nutrient scavenging [5, 12]. Concomitantly, differing assumptions on this issue have been provided and Huang et al. point out that CIC profiles may vary from tumor to tumor which may indicate different malignant stages and/or inflammatory conditions [12, 14]. On the ground of our previous findings we can support the proposition that each tumor type may harbor its own unique CIC pattern [10, 12].

Kepp et al. coined the term “immunogenic cell death” where multiple stimuli can trigger cell death that does not go unnoticed by the immune system [15]. Accordingly, immunogenic cell death is accompanied by the production of a series of immunostimulatory damage-associated molecular patterns (DAMPs), i.e. molecules that are released or exposed during cytoprotective stress responses or upon cell death [15]. Emission of DAMPs promotes the recruitment of antigen presenting cells to sites of ongoing immunogenic cell death and assists to their ability to take up dead cell-derived particulate material, as well as their capacity to prime an adaptive immune response [15]. Assuming that CICs engulf necrotic cells which would under normal circumstances release DAMPs, an immunologic reaction to the tumor could thereby be inhibited. Accordingly, occurrence of CICs as an adverse prognosticator by attenuating anti-tumoral immune responses would be coherent.

The versatile and partly antithetic observations made on CICs convey that CIC represents a highly complex form of intercellular interaction. Conceivably, various forms of CICs coexist affecting tissues and tumor progression in different ways. Elucidation of the mechanisms underlying these observations is essential to further examine the significance of cell-in-cell in healthy tissue and pathological processes.

## Conclusion

Our immunohistochemical analysis shows that CICs and senescence, taken together, occur with similar frequency in head and neck squamous cell carcinomas as apoptosis. We demonstrated that CICs have a significant impact on clinical outcome pre-RCT as opposed to senescent and apoptotic cells. We speculate that CICs impair the production of DAMPs by taking up necrotic cells inhibiting an adequate anti-tumoral immune response which is reflected by the adverse prognosis of patients with high CIC occurrences. CIC frequency might therefore be a valuable independent prognostic factor which could be taken into consideration for diagnostics and treatment strategies.

## Additional file

Additional file 1: Figure S1. Kaplan-Meier analyses. Influence of CIC structures per mm^2^, senescent cells per mm^2^ and apoptotic cells per mm^2^ in the center of the tumor of pre-therapeutic biopsies on local failure-free survival (a) and metastasis-free survival (b). Influence of apoptotic cells per mm^2^ in the center of the tumor of post-therapeutic biopsies on NED (c). (PNG 437 kb)

## Abbreviations

CIC: Cell-in-cell
CICs: Cell-in-cell structures
DAMPs: Damage-associated molecular pattern molecules
HNSCC: Head and neck squamous cell carcinoma
NCCD: Nomenclature Committee on Cell Death
NED: No evidence of disease
RCT: Radiochemotherapy
TMAs: Tissue microarrays
