# Supplementary figures and images for: Cell-in-cell structures are more potent predictors of outcome than senescence or apoptosis in head and neck squamous cell carcinomas
Source: Radiat Oncol. 2017 Jan 18;12:21. doi: 10.1186/s13014-016-0746-z (PMC5241920; doi:10.1186/s13014-016-0746-z)

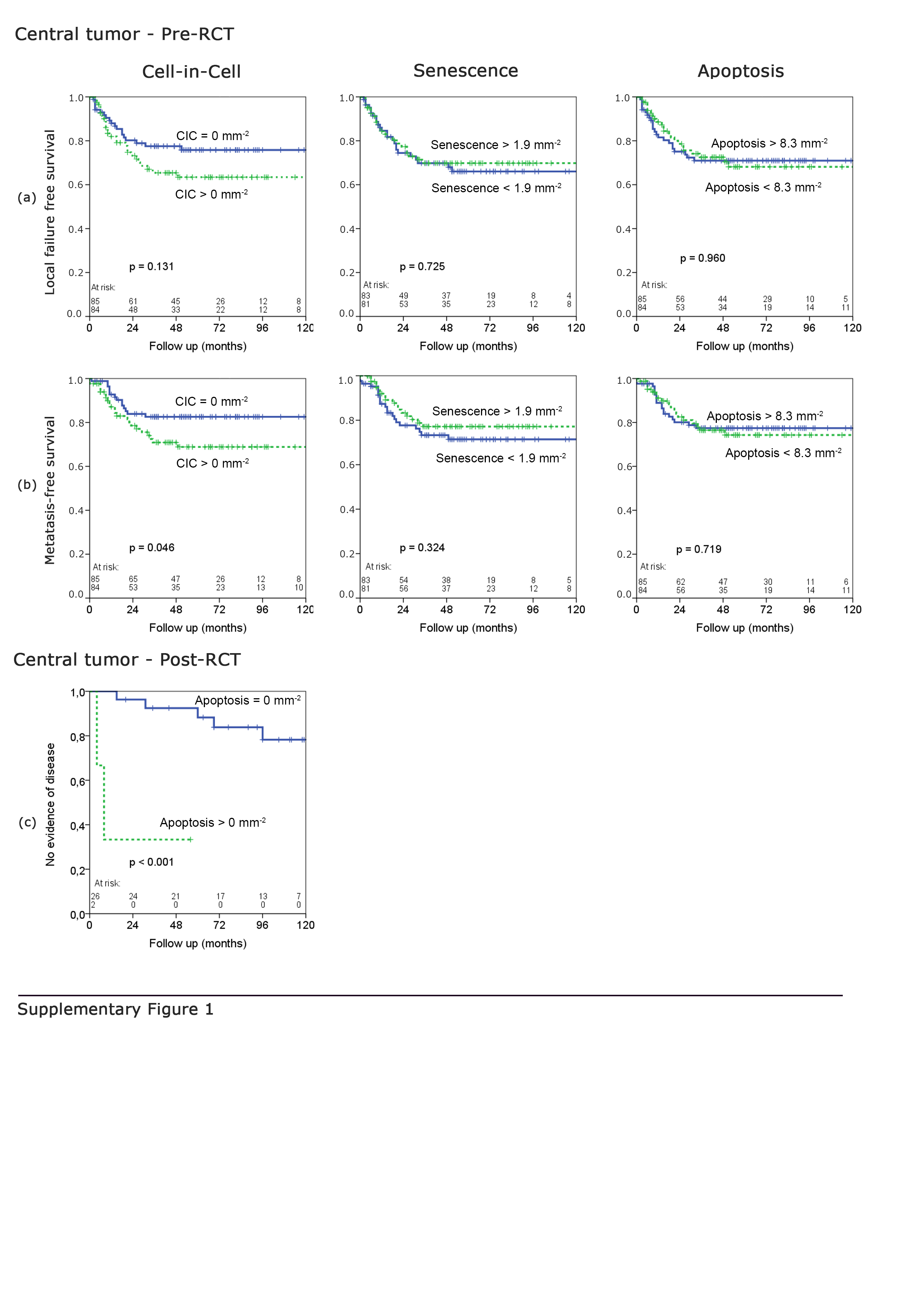

Supplement: Additional file 1: Figure S1. — Kaplan-Meier analyses. Influence of CIC structures per mm2, senescent cells per mm2 and apoptotic cells per mm2 in the center of the tumor of pre-therapeutic biopsies on local failure-free survival (a) and metastasis-free survival (b). Influence of apoptotic cells per mm2 in the center of the tumor of post-therapeutic biopsies on NED (c). (PNG 437 kb) [file 13014_2016_746_MOESM1_ESM.png]
